# Low-Density Granulocytes Are Elevated in Mycobacterial Infection and Associated with the Severity of Tuberculosis

**DOI:** 10.1371/journal.pone.0153567

**Published:** 2016-04-13

**Authors:** Yating Deng, Jianqing Ye, Qing Luo, Zhikun Huang, Yiping Peng, Guoliang Xiong, Yang Guo, Hong Jiang, Junming Li

**Affiliations:** 1 Department of Clinical Laboratory, the First Affiliated Hospital of Nanchang University, Nanchang, Jiangxi, China; 2 Department of Tuberculosis, Jiangxi Chest Hospital, Nanchang, Jiangxi, China; 3 Department of Clinical Laboratory, Jiangxi Chest Hospital, Nanchang, Jiangxi, China; University of Cape Town, SOUTH AFRICA

## Abstract

Tuberculosis remains a global health problem caused by infection with *Mycobacterium tuberculosis*. Numerous studies have established a close correlation between the development of tuberculosis and the roles of neutrophils. Recently, a distinct population of CD15^+^ granulocytes was found to be present in the peripheral blood mononuclear cell (PBMC) fraction in humans. This population of granulocytes, termed low-density granulocytes (LDGs), was reported to be elevated and associated with disease activity or severity in a number of different conditions including SLE, asthma and HIV infection. However, both the frequency and clinical significance of LDGs associated with tuberculosis are unclear. Here we determined LDG levels and made comparisons between subjects with active pulmonary tuberculosis (PTB) and healthy controls, between PTB patients with mild-to-moderate disease and patients with advanced disease, and among PTB patients following anti-tuberculous therapy of varying durations. The direct correlation between *M*. *tuberculosis* infection and LDG levels was confirmed by in vitro infection of whole peripheral blood and isolated granulocytes with mycobacteria. Our results demonstrated that PBMCs in PTB patients contained significantly elevated percentages of LDGs compared with control subjects. LDGs in tuberculosis expressed higher levels of activation markers compared to normal-density granulocytes (NDGs). *M*. *tuberculosis* induced the generation of LDGs in both whole blood and isolated NDGs from control subjects, which suggests that LDGs associated with *M*. *tuberculosis* infection are likely to originate from in situ activation. Furthermore, our results revealed that the frequency of LDGs is associated with the severity of tuberculosis.

## Introduction

Tuberculosis remains a global health problem caused by *Mycobacterium tuberculosis* infection. Numerous studies indicate that the immune response of the host critically influences the progression of *Mycobacterium tuberculosis (Mtb)* infection [1,2]; therefore, understanding the interaction between *Mtb* and the host is crucial for controlling tuberculosis.

Studies of the immune response to *Mtb* have focused primarily on T cells and macrophages, while little is known about neutrophil responses. Neutrophils are the most abundant white blood cells in humans and play crucial roles as sentinels and the first-line of defense against pathogens [3]. Research has demonstrated a characteristic neutrophil-driven transcriptional signature in the blood of patients with tuberculosis [4] and that neutrophils are the predominant cell type in both bronchoalveolar lavage fluid and sputum of patients with active pulmonary tuberculosis [5]; thus, neutrophils are implicated in immunity to *Mtb*. Furthermore, it was found, in both experimental and clinical studies, that active pulmonary tuberculosis is accompanied by massive influx of neutrophils into the lung tissues [6–8]. Although the specific role and clinical significance of neutrophils in tuberculosis are still controversial [9], these studies established a close correlation between the development of tuberculosis and infiltration of the tissues by neutrophils. However, the role of circulating neutrophils in the development of tuberculosis is largely unknown.

Recently, a distinct population of CD15^+^ granulocytes was found to be present in the peripheral blood mononuclear cell (PBMC) fraction in humans. Since these cells co-purify with PBMCs in density gradient centrifugation, this population of granulocytes was termed LDGs. These cells can be distinguished on the basis of density from normal-density granulocytes (NDGs), the other population of granulocytes that co-purify with the erythrocyte fraction. In recent years, elevated LDG levels have been reported in a number of different conditions including SLE [10], asthma [11] and HIV infection [12]. Moreover, studies demonstrate that LDG frequency is associated with disease severity in some autoimmune diseases and HIV infection [12,13]. However, at present, both the frequency and clinical significance of LDGs in tuberculosis are unclear.

In the present study, we determined the frequency and characteristics of LDGs in patients with active pulmonary tuberculosis, and conducted a preliminary investigation of the origin of LDGs in *Mtb* infection using an in vitro infection system. The correlation between LDG frequency and disease severity was also evaluated.

## Materials and Methods

### Subjects and samples

Patients with pulmonary tuberculosis (PTB) were recruited from the Jiangxi Chest Hospital and the First Affiliated Hospital of Nanchang University from April 2014 to January 2015. The TB diagnostic criteria and treatment procedures followed were in accordance with the ‘Guideline of clinical diagnosis and treatment: TB section’ of China [14]. Inclusion criteria were: patients with a confirmed diagnosis of pulmonary TB; male or female; aged between 16–65 years. Exclusion criteria were: pregnancy; comorbid conditions of HIV infection, diabetes mellitus, chronic renal failure, chronic liver disease, autoimmune diseases, and cancer. Patients with PTB were further classified as acid-fast bacillus (AFB) positive and negative according to sputum AFB microscopy, and classified as mild-to-moderate (PTB-mod) and advanced (PTB-adv) according to the extent of disease evident on chest radiography [15]. Asymptomatic healthy volunteers were recruited from April 2014 to October 2014 and assessed by tuberculin skin testing (TST) with a purified protein derivative of tuberculin. Age- and sex-matched, TST negative volunteers were selected as the control group (HC, *n* = 16) for the study.

This study was approved by the Ethical Review Committees of the First Affiliated Hospital of Nanchang University and was carried out in compliance with the Helsinki Declaration. Written informed consent was obtained from all adult subjects or guardians on behalf of the minors enrolled in this study.

### Cell isolation

Samples of peripheral blood (10 mL) were drawn by venipuncture and collected into EDTA tubes. The PBMC and NDG fractions were isolated by density gradient centrifugation. Briefly, 3 ml of venous blood was diluted 1:1 with sterile saline and was centrifuged on Lymphocyte Separation Medium (MP Biomedicals, Solon, OH, USA) in a 15-mL polystyrene conical centrifuge tube for 30 minutes at 300 ×*g* (4°C). The PBMCs were carefully collected by aspiration from the plasma-lymphocyte separation medium interface and washed once in phosphate buffered saline (PBS). When indicated, LDGs were sorted in PBMCs based on forward scatter (FSC) profile, side scatter (SSC) profile, CD14 and CD15 expression (CD14^low^ CD15^+^) by using a MOFLO cell sorter (Beckman Coulter, Brea, CA, USA).NDGs were isolated from the erythrocyte fraction by dextran sulfate sedimentation. Briefly, a quarter volumes of 6% Dextran / 0.9% NaCl solution were pipeted into the erythrocyte fraction. The mixture was pipetted into a 15-mL polystyrene conical centrifuge tube and let stand at room temperature for 45 min. The supernatant was then layered over equal volume of Ficoll-Hypaque in a new 15-mL polystyrene conical centrifuge tube and centrifuged at 1500 rpm for 30 min at 4°C. The supernatant was discarded and the cell pellet was resuspended in 2 ml PBS or RPMI 1640 medium (GIBCO, Grand Island, NY) supplemented with 10% human AB serum. All experiments were performed using fresh cells, immediately after processing. Typan Blue Staining Cell Viability Assay Kit (Beyotime Institute of Biotechnology, Jiangsu) was used to determine the cell viability in this study. Cytospin preparations were prepared and treated with Wright-Giemsa stain for morphological observation.

### Flow cytometry

The PBMCs or granulocytes (10^6^ cells each) were transferred to microcentrifuge tubes, washed once and incubated with FcR blocking reagent (Miltenyi Biotec, Auburn, CA, USA) for 5 min. The cells were then incubated for 30 min at room temperature without or with the following mixture of fluorescent-labeled anti-human monoclonal antibodies: CD14-ECD, CD15-PE-Cy5 (Beckman Coulter); CD16-FITC, CD33-FITC, CD66b-FITC, CD62L-FITC (eBioscience, San Diego, CA, USA). Cell doublets were eliminated based on the size of the event (FSC). Analysis was performed by using a Cytomics FC 500 flow cytometer (Beckman Coulter, Brea, CA, USA).

### Bacterial strains and infection

*Mtb* H37Rv strain, *M*. *bovis* BCG Danish strain, *M*. *smegmatis* Mc2-155 strain and *Mtb*-GFP were grown to early mid-log phase in Middlebrook 7H9 broth, supplemented with 10% albumin-dextrose-catalase and 0.5% glycerol at 37°C. The bacterial aggregates were shattered by gentle agitation with 3-mm-diameter glass beads. The resultant bacteria were diluted in PBS. The solution was left standing for 15 min before the supernatant was collected and adjusted to an OD_600_ of 0.5 (approximately 10^7^ individual bacteria/mL).

For in vitro infection, whole blood or purified NDGs were infected by mycobacteria at the multiplicity of infection (MOI) of 5 (bacteria to neutrophils) for indicated times. Following the infection of whole blood, discontinuous density gradient centrifugation was performed and the LDGs and NDGs were counted by flow cytometry using aforementioned methods. Following the infection of purified NDGs, cells were washed once and resuspended in 3 ml sterile saline, then added to 3 ml of autologous uninfected whole blood. LDGs in this mixture and uninfected venous blood were counted by flow cytometry following discontinuous density gradient centrifugation using aforementioned methods. PI positive dead cells were excluded by PI staining. The number of LDGs in infected neutrophils was calculated by subtracting the number of LDGs in uninfected blood with LDGs in the mixture. All performance involving bacterial handling was performed in class Ⅱ biological safety laboratory.

### Measurement of reactive oxygen species

The levels of intracellular reactive oxygen species (ROS) were measured using the oxidation-sensitive fluorescent dye H_2_DCF-DA (2′,7′-dichlorodihydrofluorescein diacetate; Invitrogen, Carlsbad, CA, USA). Briefly, cells were loaded with 10 μM H2DCF-DA (Invitrogen) for 30 min at 37°C in the dark, then washed once with PBS and analyzed immediately for ROS levels by flow cytometry (FCM).

### Phagocytosis and bactericidal characteristics assay

LDGs and NDGs (1×10^6^ cells) were plated in 24-well plates in RPMI 1640 medium supplemented with 10% human AB serum and incubated at 37°C for 1 h in a humidified incubator with 5% CO_2_. To determine the phagocytic capacity of neutrophils, *M*.*tuberculosis* -GFP was added to the cell cultures at the multiplicity of infection (MOI) of 5. After 30 min of incubation, cells were washed thoroughly with D-Hanks buffer to remove extracellular bacteria and analyzed by using Cytomics FC 500 flow cytometer (Beckman Coulter Inc., Brea, CA, USA). To determine the bactericidal activity of neutrophils, *M*.*tuberculosis* was incubated with (at the MOI of 1) or without neutrophils. Neutrophils were lysed with 0.01% SDS at indicated time points and serial 10-fold dilutions of each lysed sample were plated on Middlebrook 7H10 agar plates. Colonies were counted after three-week incubation.

### Statistical analysis

Data were evaluated for statistical differences using Student *t* test, one-way ANOVA test with pos-hoc analysis and Kruskal-Wallis test when appropriate (GraphPad Prism 5); Differences were considered statistically significant at *P* < 0.05. Unless indicated otherwise, results are expressed as mean±SEM.

## Results

### Characteristics of study subjects

The study population comprised 67 subjects with PTB (mean age 50.2±16.2 years) and 16 control subjects with no history of tuberculosis (mean age 47.1±10.6 years). Of all the recruited patients with PTB, 31 subjects (NATT-PTB) were newly diagnosed and had not received anti-tuberculous therapy (ATT); 23 subjects (ATT-2w-PTB) had received 2 weeks of ATT in hospital and 13 subjects (ATT-6m-PTB) had received a further 6 months of ATT at home following 2 weeks of hospitalization. No multi-drug resistant tuberculosis patient was included in this study and all patients who received ATT showed a good response to treatment. Newly diagnosed PTB patients comprised 20 subjects with mild-to-moderate disease and 11 subjects with advanced disease.

### Elevated LDG in peripheral blood of patients with PTB

**Fig 1:**
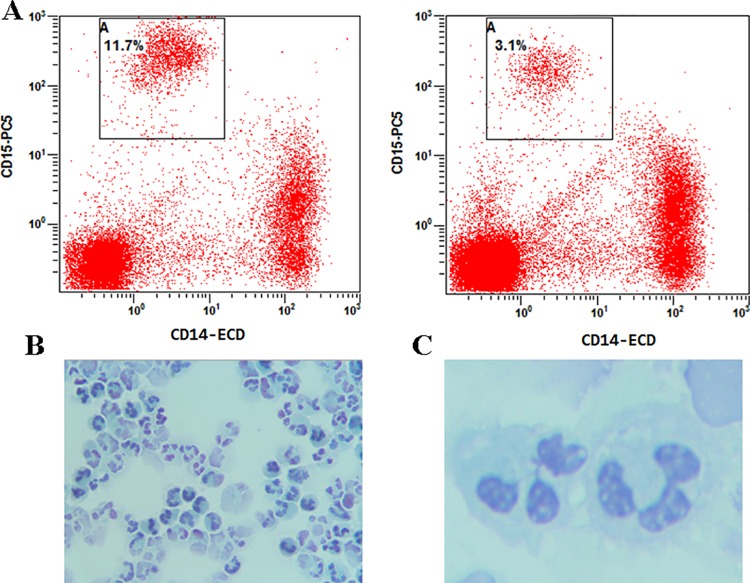
Flow cytometric (FCM) analysis of low-density granulocytes (LDGs) in peripheral blood mononuclear cells (PBMCs). PBMCs were isolated by Ficoll gradient centrifugation from peripheral blood samples of subjects. LDGs were gated in PBMCs as CD14^low^ CD15^+^ populations by flow cytometry (A) and confirmed by morphological observation after Wright-Giemsa staining (B: ×400, C: ×1,000). Each image shows a single subject and the relative percentage of LDGs is displayed.

The LDGs were identified in PBMCs as CD14^low^ CD15^+^ granulocytes as described in previous reports [10,12]. Indeed, the monocytes and LDGs could be distinguished clearly based on expression of the neutrophil marker CD15 and the monocyte marker CD14 (Fig 1A). Wright-Giemsa staining showed that all cells in this population (>95%) displayed typical neutrophil morphology (Fig 1B and 1C).

**Fig 2:**
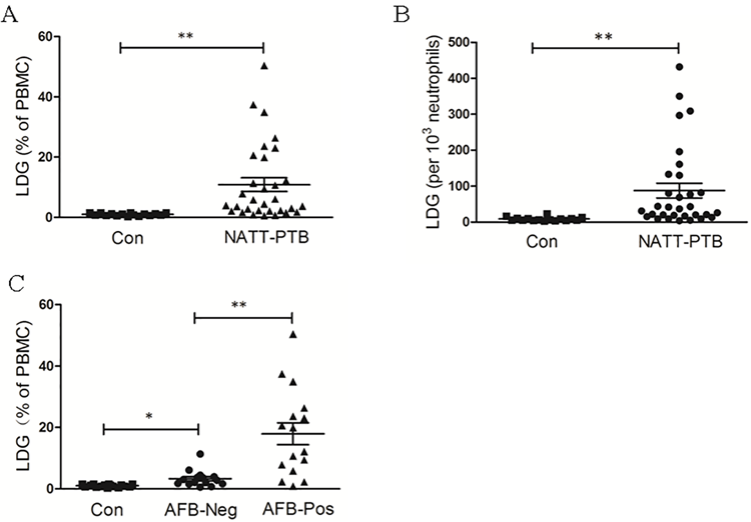
Levels of LDGs in the PBMC fraction of healthy controls (Con) and pulmonary tuberculosis (PTB) patients. (A) The percentages of LDGs in PBMC fraction and (B) the number of LDGs in PBMC fraction per 10^3^ neutrophils in the erythrocyte fraction of control subjects and PTB patients who had not received anti-tuberculous therapy (NATT-PTB) are shown. Each symbol denotes a single subject, and the mean ± standard error of the mean (SEM) for each study population is shown. Statistical significance was determined by a Student *t* test. (C) NATT-PTB subjects were then further divided into two groups based on the results of sputum acid-fast bacillus (AFB) staining, and the percentages of LDGs in PBMC fraction in each group are shown. Each symbol denotes a single subject, and the mean ± SEM for each study population is shown. Statistical significance was determined by a one-way ANOVA followed by the Gabriel post hoc test. *P < 0.05, **P < 0.01.

Our results showed that the PBMCs of subjects with NATT-PTB contained significantly elevated percentages of LDGs compared with the PBMCs of control subjects (10.84±8.68% versus (vs.) 0.99 ±0.40%, *P* < 0.01; Fig 2A). The ratio of the number of LDGs in the PBMCs to 10^3^ NDGs isolated in the erythrocyte fraction was also significantly elevated in subjects with NATT-PTB (Fig 2B). Further analysis demonstrated that the percentages of LDGs in the PBMCs of newly diagnosed patients with positive AFB were significantly elevated compared with those of patients with negative AFB (17.95±11.27% vs. 3.26 ±2.67%, *P* < 0.01; Fig 2C). This suggested that LDG levels are elevated in the peripheral blood of patients with PTB, and may be correlated with bacterial burden.

### Characteristics of LDGs in tuberculosis

**Fig 3:**
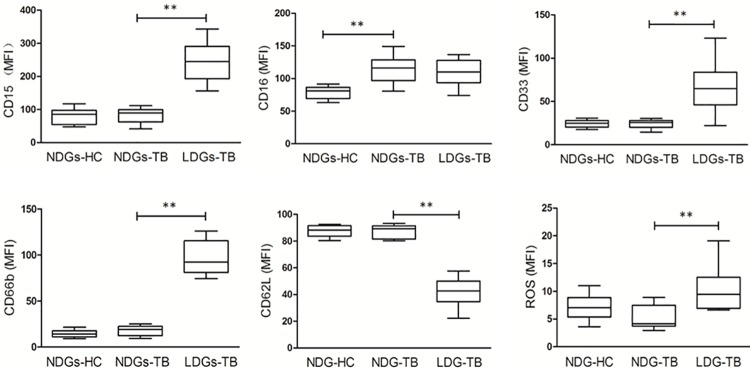
Detection of selected surface CD molecules and ROS generation in LDGs and NDGs. LDGs and NDGs from patients with tuberculosis (TB) and healthy control subjects (HC) were analyzed by FCM for the expression levels of CD15, CD16, CD33, CD66b and CD62L, and the amounts of ROS. Statistical significance was determined by a kruskal-Wallis test. Box = interquartile range and median; whiskers = range. **P < 0.01.

Previously studies have shown that LDGs in patients with SLE and HIV infection are phenotypically different from NDGs [10–12]. Therefore, the expression levels of phenotypic markers on the surface of LDGs and autologous NDGs were compared to further confirm the identity of LDGs in patients with PTB. Data showed that, when compared to NDGs-TB (NDGs from patients with PTB), the mean fluorescence intensities (MFI) of membranal CD15, CD33 and CD66b were significantly enhanced on autologous LDGs-TB (LDGs from patients with PTB). The levels of membranal CD62L were significantly reduced on LDGs-TB compared with those of NDG-PTB and NDGs-HC (NDGs from subjects of health control). ROS production was also determined and compared among LDGs-TB, NDGs-TB and HC-NDGs. Data showed that LDGs-TB produced significantly increased amounts of ROS compared to NDGs-TB and NDGs-HC. No significant difference was observed in the MFI of CD16 staining between LDGs-TB and NDGs-TB. With the exception of CD16, no difference in the levels of all surface phenotypic markers was found between NDGs-TB and NDGs-HC (Fig 3, S1 Table).

**Fig 4:**
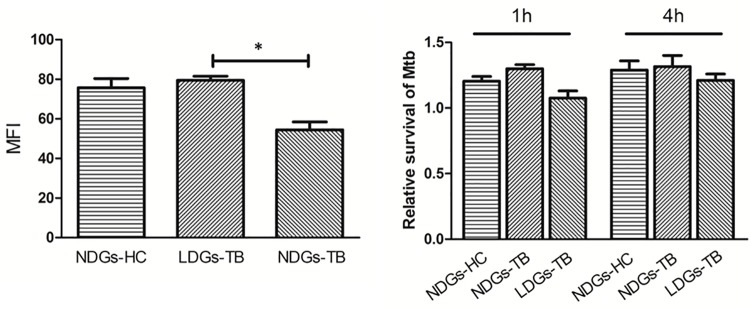
Phagocytic capacity and bactericidal activity of LDGs and NDGs. (A) LDGs and NDGs from patients with tuberculosis (TB) and healthy control subjects (HC) were isolated as described in materials and methods and infected with *M*.*tuberculosis*-GFP at the multiplicity of infection (MOI) of 5. Phagocytosis of bacteria was detected by flow cytometry at 30 min postinfection. Results represent mean ± SEM of mean fluorescence intensity of infected neutrophils. (B) *M*. *tuberculosis* H37Rv was incubated with (at the MOI of 1) or without neutrophils for indicated times and the colony forming units (cfu) were monitored over times. Results represent the ratios of cfu in LDGs and NDGs to cfu in cultures without neutrophils at indicated time points. Significance was determined by by one-way ANOVA followed by the Tukey post hoc tes. *P < 0.05.

Next, the phagocytosis and bactericidal effects of LDGs were examined by flow cytometry and colony forming unit (CFU) assay, respectively. Data showed that LDGs from TB patients displayed significantly increased phagocytosis of *M*.*tuberculosis* when compared to autologous NDGs, but comparable phagocytosis of *M*.*tuberculosis* when compared to NDGs from healthy control (Fig 4A). CFU assays demonstrated that both LDGs and NDGs cannot kill M.tuberculosis. However, the bacterial load in LDGs from TB patients was somewhat decreased when compared to NDGs from healthy control or TB patients, but the difference was not statistically significant (Fig 4B, S2 Table).

### Mtb infection induces the generation of LDGs in dose- and time-dependent manner

**Fig 5:**
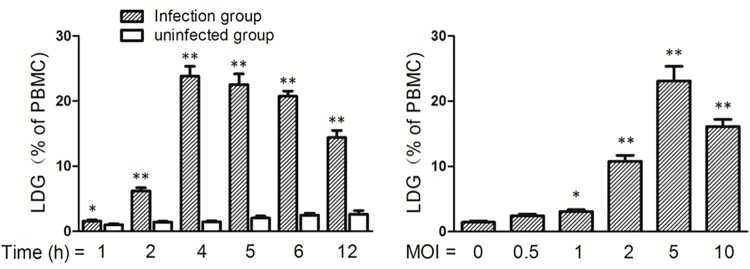
Levels of LDGs following in vitro infection with *M*. *tuberculosis*. Peripheral blood samples were collected from healthy volunteers and challenged with *M*. *tuberculosis* H37Rv (A) for the indicated times at the MOI of 5 or (B) at the indicated MOI for 4 h. The percentages of LDGs in PBMC fraction were determined by FCM following discontinuous density gradient centrifugation. Data represent the mean ± SEM of at least three repeated measures tests from a single subject. Significance was determined by comparison with the uninfected group by student *t* test (A) and one-way ANOVA followed by the Tukey post hoc test (B). *P < 0.05, **P < 0.01.

The previously described results demonstrated that patients with PTB have significantly elevated levels of LDGs in their peripheral blood, suggesting that *Mtb* induces the generation of LDGs, or promotes the conversion of NDGs to LDGs. To confirm the correlation of *Mtb* infection with the elevated level of LDGs, peripheral blood was collected from asymptomatic healthy volunteers, challenged in vitro with *Mtb* H37Rv for indicated time, subjected to density centrifugation and analyzed for the levels of LDGs by FCM. Results showed that the levels of LDGs were significantly elevated following *Mtb* infection in a dose- and time-dependent manner (Fig 5).

**Fig 6:**
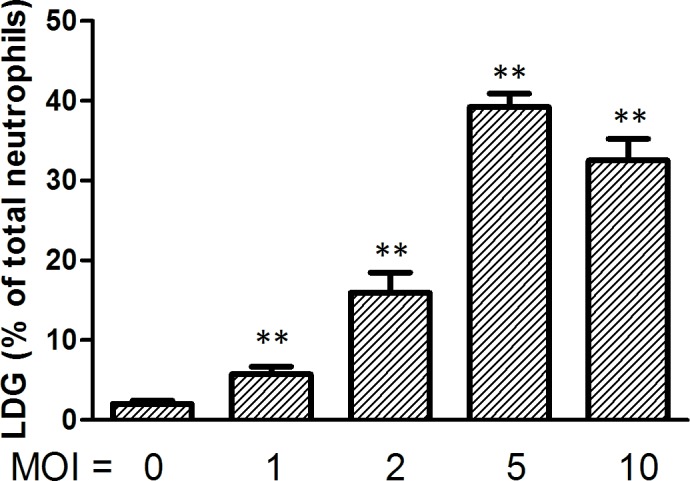
*M*. *tuberculosis* converts normal-density granulocytes (NDGs) to low-density granulocytes (LDGs). Neutrophils were isolated from the erythrocyte fraction of healthy volunteers and in vitro challenged with *M*. *tuberculosis* H37Rv at the indicated MOI for 4 h. Cells were then washed once, resuspended in 3 ml sterile saline and added to 3 ml of autologous uninfected whole blood. LDGs in this mixture and uninfected venous blood were counted by flow cytometry following discontinuous density gradient centrifugation. The number of LDGs in infected neutrophils was calculated by subtracting the number of LDGs in uninfected blood from LDGs in the mixture, and the percentages of LDGs to total infected neutrophils were calculated and displayed. Data are expressed as mean ± SEM. Significance was determined by comparison with the uninfected group by a one-way ANOVA followed by the Tukey post hoc test. **P < 0.01.

In addition, neutrophils were isolated from the erythrocyte fraction of peripheral blood from asymptomatic healthy volunteers and challenged in vitro with *Mtb* H37Rv. The generation of LDGs was detected by FCM after discontinuous density gradient centrifugation over Ficoll-Hypaque. Data showed that *Mtb* infection induced the generation of LDGs in isolated NDGs. In accordance with the results observed following infection of whole blood, the levels of LDGs were related to the MOIs used for infection (Fig 6). These results suggested that *Mtb* infection converts NDGs to LDGs in a dose-dependent manner.

Characterization of the expression of surface molecules indicated that the LDGs derived from in vitro challenge with *Mtb* express elevated levels of CD15 and CD66b, similar levels of CD16 and CD33, and decreased levels of CD62L compared with NDG. The intracellular levels of ROS were also increased in LDGs compared to those in NDGs following *Mtb* infection (data not shown). With the exception of the expression of CD33, all these phenotypic characteristics of LDGs derived from in vitro challenge with *Mtb* are consistent with those of LDGs in patients with tuberculosis.

### LDG induction is associated with mycobacterial virulence properties and metabolic activity

**Fig 7:**
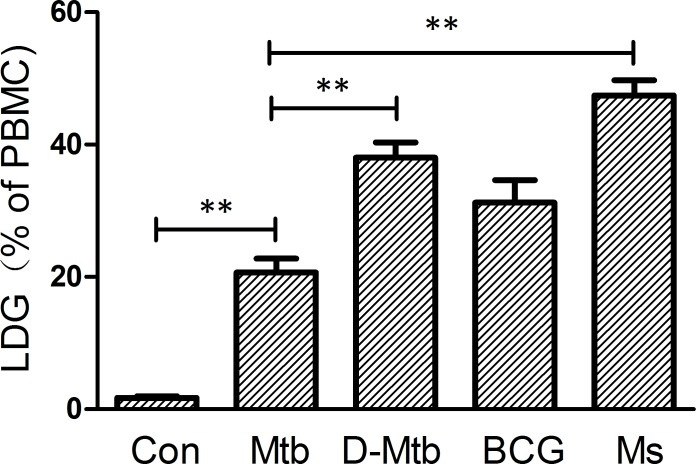
Levels of low-density granulocytes (LDGs) following mycobacterial infection. Peripheral blood samples were collected from healthy volunteers and challenged in vitro with *M*. *tuberculosis* H37Rv, heat-killed *M*. *tuberculosis* H37Rv, *M*. *bovis* BCG or *M*. *smegmatis* Mc2-155 at a MOI of 5 (bacilli to neutrophil number in whole blood) for 4 h. The percentages of LDGs in PBMC fraction were then determined by flow cytometry following discontinuous density gradient centrifugation. Data represent the mean ± SEM of at least three repeated measures tests from a single subject. Significance was determined by comparison with the uninfected group by a one-way ANOVA followed by the Tukey post hoc test. **P < 0.01.

Previous studies and our results have suggested that LDGs are activated neutrophils that have degranulated. It is well-known that compared to avirulent mycobacteria, virulent *Mtb* can moderate neutrophil activation [16,17]. To investigate the potential correlation between mycobacterial virulence and the capacity to induce LDG generation, peripheral blood was collected from asymptomatic healthy volunteers, challenged with *M*. *smegmatis* Mc2-155, *M*. *bovis* BCG, and live or heat-killed *Mtb* H37Rv, respectively. The percentage of LDGs in the PBMC fraction was determined by FCM (Fig 7). Data demonstrated that infection with *M*. *bovis* BCG, a slow-growing avirulent vaccine strain of mycobacteria, induced significantly more LDGs than virulent *Mtb* H37Rv (31.22±4.33 vs. 20.68±3.63, *P* < 0.05), but induced significantly reduced LDGs compared to *M*. *smegmatis*, a fast-growing avirulent mycobacteria (31.22±4.33 vs. 47.40±5.58, *P* < 0.05). Furthermore, we found that the frequency of LDGs induced by live *Mtb* was significantly decreased compared to heat-killed *Mtb* (20.68±3.63 vs. 38.04±4.74, *P* < 0.01), suggesting that the metabolic activity of *Mtb* inhibits LDG generation.

### Association between LDG Levels and severity of tuberculosis

**Fig 8:**
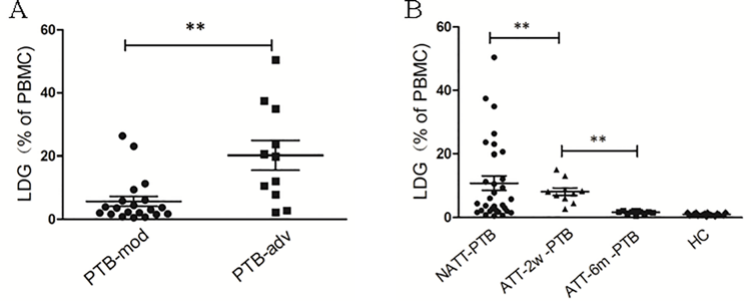
Frequency of LDGs in the PBMCs of PTB patient correlated with disease severity. (A) PBMCs from PTB patients with mild-to-moderate disease (PTB-mod) and advanced disease (PTB-adv) were isolated by Ficoll gradient centrifugation and the percentages of CD14low CD15+ LDGs were determined by FCM. Each symbol denotes a single subject, and the mean ± SEM for each study population is shown. Statistical significance was determined by a Student *t* test. (B) PBMCs from healthy control subjects, PTB patients who had not received anti-tuberculous therapy (NATT-PTB), and those who had received 2 weeks of ATT in hospital (ATT-2w -PTB) or 6 months of ATT in the home following 2 weeks of hospitalization (ATT-6m -PTB) were isolated by Ficoll gradient centrifugation and the percentages of CD14low CD15+ LDGs were determined by FCM. Each symbol denotes a single subject, and the mean ± SEM for each study population is shown. Statistical significance was determined by a one-way ANOVA followed by the Gabriel post hoc test. **P < 0.01.

The previously described results demonstrated a correlation between the frequency of LDGs and the multiplicity of mycobacterial infection. To assess the potential clinical significance of this phenomenon, we analyzed the correlation between the frequency of LDGs and the severity of tuberculosis. Patients with active PTB were recruited and classified as mild-to-moderate disease (PTB-mod) and advanced disease (PTB-adv) according to the extent of disease evident on chest radiography; these patients were analyzed for the frequency of LDGs in the PBMC fraction. The levels of LDGs in patients with PTB-adv were significantly higher than those in patients with PTB-mod (Fig 8A). Furthermore, we compared the frequency of LDGs in PTB patients who had not received TAA and those who had different durations of TAA. As shown in Fig 8B, the percentages of LDGs in the PBMCs of PTB patients following 2w-TAA were significantly lower than those in patients who had not received TAA. After 6-months of ATT, the percentage of LDGs in PTB patients recovered to the levels of those in healthy volunteers. These results demonstrated that in patients with PTB, the frequency of LDGs in the PBMC fraction correlated with disease severity.

## Discussion

The results reported here provide an insight into several aspects of LDGs in tuberculosis. First, the levels of LDGs were significantly elevated in tuberculosis. Compared to autologous NDGs, the LDGs found in the present study expressed elevated levels of CD15, CD33 and CD66b, decreased levels of CD62L and similar levels of CD16. Furthermore, the LDGs in tuberculosis patients were found to contain significantly higher amounts of ROS. The ROS production and the profile of surface molecule expression (with the exception of CD33), suggest that LDGs in tuberculosis are activated neutrophils that have degranulated. Thus, LDGs in tuberculosis display an activated or primed neutrophil phenotype, which is similar to that of the LDGs described previously in patients with autoimmune diseases and those with HIV infection [12,13,18]. In addition, our study shows for the first time that in vitro infection with *Mtb* induces the generation of LDGs.

Second, we found that the generation of LDGs following *Mtb* infection is dose- and time-dependent. Furthermore, our study showed that avirulent mycobacteria are more powerful than virulent *Mtb* in inducing the generation of LDGs, and that the levels of LDGs induced by live *Mtb* were significantly decreased compared to the levels induced by heat-killed *Mtb*. These results demonstrate that live *Mtb* inhibits the production of LDGs to some extent via a mechanism that is related to its virulence and metabolic activity.

Third, we found that the levels of LDGs in patients with advanced tuberculosis were significantly higher than those in patients with mild-to-moderate disease and that ATT significantly decreased the percentage of LDGs in PTB patients. These results revealed a correlation between LDG levels and the severity of tuberculosis. Although the contribution of these elevated levels of LDGs to the severity of tuberculosis remains to be determined, this preliminary study does highlight the potential of elevated levels of LDGs to serve as a biomarker of tuberculosis severity.

Recent evidence suggests that LDGs contribute to tissue damage in some autoimmune diseases [13,19,20]. In accordance with these reports, a correlation between the frequency of LDGs and the severity of tuberculosis was observed in the present study, which suggested that LDGs might play a pathogenic role in tuberculosis.

However, we also found that when compared to avirulent and dead mycobacteria, virulent *Mtb* inhibited the generation of LDGs to some extent. Combining with the elevated expression of activation markers and increased intracellular ROS in LDGs, this finding made us speculate that LDGs might possess more powerful potential in killing Mtb. However, our results revealed that LDGs can not kill *Mtb*, which is similar to NDGs in this study and neutrophils in reported researches [21].

Previous studies indicate several possibilities concerning the origin of LDGs. On the basis of high expression levels of CD33, a marker of immature neutrophils [22], some studies proposed that LDGs arise as the consequence of disruption of granulocyte development [22,23]. However, characterization of the surface molecule expression and nuclear morphology conducted as part of this and other studies [10] indicate that LDGs are a population of fully mature granulocytes. Besides functioning as a marker of developing or immature granulocytes, CD33 is also involved in adhesion and migration of neutrophils [24]. Thus, we hypothesize that the increased expression of CD33 represents an elevation in the adhesion or migration capacity of LDGs; however, this requires confirmation in further studies. Based on studies in SLE, it has also been speculated that LDGs are derived by in situ activation of NDGs. Results of the present study indicate that LDGs are activated neutrophils that have degranulated. Moreover, results from the in vitro infection studies demonstrated that *Mtb* can rapidly convert NDGs to LDGs. Furthermore, the profile of LDGs derived from in vitro challenge with *Mtb* is very similar to that of the LDGs in patients with tuberculosis. Thus, these results reveal that in situ activation contributes to the generation of LDGs in tuberculosis.

There are, of course, some limitations to this present study, including the fact that the blood samples from TB patients receiving no ATT or ATT of varying duration were not collected from the same individuals in a paired manner. Patient variability may also affect the analysis of our results. Furthermore, because of the limitation of research subjects, we did not assess the LDG levels in patients who did not respond to ATT. Nonetheless, the striking consistency in the levels of LDGs observed among the different groups investigated suggests that this difference is meaningful.

In summary, the present study provides the first evidence of activated LDG levels in the PBMC fraction of peripheral blood in patients with active pulmonary tuberculosis, established a correlation between the frequency of LDGs and the severity of tuberculosis, and revealed the possible origin of LDGs following *Mtb* infection. Nonetheless, further investigations are required to elucidate the clinical significance of the elevated levels of LDGs in tuberculosis. The signals resulting in activation of neutrophils and their development into LDGs following *Mtb* infection also remain to be clarified.

## Supporting Information

S1 Table Detection of selected surface CD molecules and ROS generation in LDGs and NDGs. LDGs and NDGs from patients with tuberculosis (TB) and healthy control subjects (HC) were analyzed by FCM for the expression levels of CD15, CD16, CD33, CD66b and CD62L, and the amounts of ROS (MFI).
(XLSX)

S2 Table Replication of *M*.*tuberculosis* incubated with or without neutrophils. *M*. *tuberculosis* H37Rv was incubated with (at the MOI of 1) or without neutrophils for indicated times and the colony forming units (cfu) were monitored over times.
(XLSX)
